# Research highlights, *The Plant Genome*, Volume 14, Issue 2

**DOI:** 10.1002/tpg2.20137

**Published:** 2021-07-19

## 1 SYSTEMS BIOLOGY FOR CROP IMPROVEMENT

Recent technological innovations have enabled generation of high‐resolution datasets from different levels of biological organization. This has led to an array of approaches carrying the ‘omics’ suffix such as genomics, pangenomics, transcriptomics, proteomics, metabolomics, epigenomics, and, more recently, single‐cell omics, phenomics, and QTLomics. Systems biology has recently emerged as a promising multidisciplinary research field that integrates large omics datasets coupled with well‐designed mathematical models to confirm hypotheses and predict biological systems. This provides a more holistic understanding of system‐wide response during growth, development, and stress adaptation in crops. Thus, Pazhamala et al. (https://doi.org/10.1002/tpg2.20098) report that systems biology provides great potential for sustainable agriculture by understanding the complexity of multiple traits bridging the genotype–phenotype gap, crucial for next‐generation breeding of climate‐ready crops.

## 2 A GENOMICS RESOURCE FOR GENETICS, PHYSIOLOGY, AND BREEDING OF WEST AFRICAN SORGHUM

Sorghum is one the world's most climate‐resilient crops, but African plant breeders are looking for new tools to adapt sorghum for farmer and consumer needs. Faye et al. (https://doi.org/10.1002/tpg2.20075) report the development of a genomic toolkit for West African sorghum that captures diversity of farmer‐preferred varieties and four national breeding programs. They gained insight into the genetic relationships of traditional varieties and breeding lines and identified key genes underlying yield and adaptation across sorghum breeding programs in western Africa where the crop is a staple food security cereal for millions of people. This genomic resource provides a foundation for genomics‐enabled breeding of climate‐resilient varieties in sub‐Saharan Africa.

## 3 MAPPING SEED QUALITY TRAITS IN *BRASSICA* C GENOME

Increasing seed oil and protein contents and reducing the content of seed glucosinolates (GSLs) in oilseed rape or canola (*Brassica napus*) are important objectives in breeding. By using an oilseed rape population carrying genome content introgressed from Chinese kale (*Brassica oleracea*, a parental species of oilseed rape), Rahman and Kebede (https://doi.org/10.1002/tpg2.20078) identified a stable QTL for seed oil content on chromosome C5 at 40–42 Mb position and a stable QTL for seed GSL content on C9 at 7–8 Mb position; the C5 and C9 QTL alleles for high oil and high GSL contents derived from Chinese kale. These two QTL did not exert a significant effect on days to flowering and seed yield. However, the low‐GSL QTL allele of C9 exerted a significant positive effect on seed protein content demonstrating that selection for this allele contributed to higher protein content in canola seed. Thus, the genomic regions and the molecular markers identified in this study should be useful in molecular breeding of the seed quality traits in oilseed rape/canola. The results from this study also demonstrate that desirable alleles for seed quality traits can be found in the parental species of canola for use in the breeding of this oilseed crop.

## 4 GENOMIC SELECTION DRIVES PERENNIAL GRAIN FORWARD

Perennial grains could provide substantial ecosystem services and food for humans but currently there are no widely available, high‐yielding perennial grains for annual grain crop substitutes. Crain et al. (https://doi.org/10.1002/tpg2.20080) report on the genetic gains of intermediate wheatgrass (*Thinopyrum intermedium*, trade name Kernza) using genomic selection. By incorporating genomic selection into the breeding program, cycle time was cut in half and up to 8% increase in genetic gain per year could be achieved for grain yield. These rapid genetic increases will support the domestication and utilization of intermediate wheatgrass as a perennial grain crop.

## 5 NOVEL GENE INTERACTION CONFERS HIGH RESISTANCE TO BOTH RENIFORM AND CYST NEMATODES IN SOYBEAN

Reniform nematode (RN) is among the most damaging plant parasitic nematodes of soybean. Crop rotation with resistant soybean cultivars is the best recommended management tool; however, there are very few soybean cultivars with a high level of RN resistance. In the current study, Usovsky et al. (https://doi.org/10.1002/tpg2.20083) identified two RN resistance regions in soybean; one genomic region contains a known soybean cyst nematode (SCN) resistance gene, the *GmSNAP18* (*rhg1‐a*), and another one contains its related paralog (*GmSNAP11*). Interestingly, both genes seem to play a role in resistance to both RN and SCN. Moreover, the combination of resistant alleles from *GmSNAP18* and *GmSNAP11* provides high resistance to both nematodes.

## 6 SELECTION REDUCES GENETIC VARIATION IN LETTUCE

Genetic diversity is essential in crop breeding to improve cultivars with desirable traits. Park et al. (https://doi.org/10.1002/tpg2.20086) provides evidence that selective breeding for delayed bolting led to the reduction of genetic variance in crisphead lettuce. Among four horticultural types of lettuce—butterhead, crisphead, leaf, and romaine—the crisphead type lettuces were the most homogenous with the slowest linkage disequilibrium decay. The genome regions with low genetic variation, so called selective sweeps, largely overlapped with loci associated with delayed bolting in the crisphead lettuces. Thus, the positive selection of delayed bolting appeared to contribute to the reduced variation, and in future breeding, the crisphead type would require targeted introgression and larger population sizes.

## 7 GENETICS OF FIRE BLIGHT RESISTANCE IN APPLE

Fire blight is the most destructive bacterial disease of apple (*Malus* spp.). Thapa et al. (https://doi.org/10.1002/tpg2.20087) used a diverse set of 566 apples and identified novel variations in the genome of apples underlying fire blight resistance. There was large variation in fire blight resistance and susceptibility in these apples for both shoot and blossom fire blight. Interestingly, some of the genomic variations linked to fire blight resistance are colocalized with previously identified resistance to fire blight or apple scab. In addition, candidate genes for fire blight resistance were identified within these genomic intervals. Future validation and development of diagnostic DNA markers linked to these genomic variations underlying fire blight resistance will facilitate breeding apple cultivars with decreased fire blight susceptibility.

## 8 MODELLING FIRST ORDER ADDITIVE × ADDITIVE EPISTASIS IMPROVES ACCURACY OF GENOMIC PREDICTION FOR SCLEROTINIA STEM ROT RESISTANCE IN CANOLA

Sclerotinia stem rot, caused by the fungus *Sclerotinia sclerotiorum*, is a recalcitrant issue to canola (*Brassica napus*) growers worldwide. However, there are very few commercial canola varieties with resistance to sclerotinia stem rot available, most likely due to the complex genetic architecture of this trait. Derbyshire et al.  (https://doi.org/10.1002/tpg2.20088) applied genomic prediction, a process used to link whole‐plant genotype to phenotype, to this trait in a population of 218 *B. napus* plants. Increases in breeding value prediction accuracy were observed for models that incorporated a statistical term for genetic interactions (epistasis), indicating that these models were a better fit for the trait architecture. This could be useful for designing breeding strategies for sclerotinia stem rot resistance in canola.

## 9 BREEDING PERENNIAL GRAIN WITH GENOMIC SELECTION

Perennial grains could greatly increase agricultural sustainability, but a key challenge of breeding perennial crops is the long breeding cycle. Crain et al. (https://doi.org/10.1002/tpg2.20089) report methods to implement genomic selection for breeding intermediate wheatgrass (*Thinopyrum intermedium*, trade name Kernza). By leveraging next‐generation sequencing technology and historical evaluation data, breeding cycle time could be cut in half, potentially doubling genetic gains. Importantly, plants could be evaluated for multiple years while still making crosses every year, providing a tractable way to breed for long‐term traits like sustained yield, while not slowing the breeding cycle. Use of genomic selection will aid in the rapid domestication of intermediate wheatgrass as a perennial grain crop and provide a framework for new crop domestication.

## 10 *LATHYRUS* MLO1 CHARACTERIZATION AND MAPPING

Powdery mildews are major diseases for a range of crops. The loss of function of specific *Mildew Locus O* (*MLO*) genes has long been associated with plant resistance to powdery mildew and has proven to be durable in several species. Santos et al. (https://doi.org/10.1002/tpg2.20090) isolated and characterized *MLO1* genes in *Lathyrus sativus* and *L. cicera* genotypes with varied levels of partial resistance against powdery mildew. Phylogenetic analyses confirmed that *Lathyrus* MLO1 belongs to Clade V, like all dicot MLO proteins associated with powdery mildew susceptibility. DNA sequence polymorphisms between the analyzed genotypes allowed the location of MLO1 in the newly developed *L. sativus* genetic linkage map. Subsequent comparative mapping between *L. sativus* and *L. cicera* genetic maps and *Pisum sativum*, *Lens culinaris* Medik., and *Medicago truncatula* Gaertn. reference genomes revealed important aspects of the conservation of the *MLO1* locus position and of the overall chromosomal rearrangements occurring during legume evolution, with relevance to legume disease resistance breeding programs.

## 11 SUSCEPTIBLE SOYBEAN CONTRIBUTES TO NEMATODE RESISTANCE

Soybean cyst nematode (SCN) resistance in soybean is a multigenic trait with more than 300 quantitative trait loci (QTL) reported. Huang et al. (https://doi.org/10.1002/tpg2.20091) discovered that even susceptible soybean contributed multiple loci to nematode resistance by using chromosome segment substitution lines which were confirmed as valuable sources for breeding program and powerful genetic analysis tools to determine QTL effects for resistance, especially for minor QTL effect. This is the first time a new evaluation method of nematode reproduction with cysts per gram root was introduced and the associated QTL were mapped. The combination of all positive alleles for SCN female index and cysts per gram root exhibited low nematode reproduction to produce transgressive resistance. Thus, except SCN female index, cysts per gram root should be considered for breeding purpose and transgressive inheritance is an important way to obtain novel or enhanced levels of pathogen resistance for crop improvement.

## 12 HEAT‐SHOCK PROTEINS HELP WHEAT FIGHT PATHOGEN

Heat‐shock proteins are a class of important molecular chaperones and play crucial roles in plant development and adaptability to stresses. Guo et al. (https://doi.org/10.1002/tpg2.20092) systematically classified heat‐shock family proteins of wheat and uncovered the diversity and characterization, especially those involved in protecting plants from fungal disease. Infection by powdery mildew and stripe rust pathogen triggered robust alteration in heat‐shock protein‐encoding genes expression in wheat, but the regulated model varied in response to the two kinds of pathogen. TaHsp70‐30b protein interacts with heat shock transcription factor A9‐like protein and TaHsp90‐4b. This hinted that heat shock proteins played important roles in wheat fighting fungi by a multi‐chaperone integrated pathway.

## 13 A STEPWISE EVOLUTION OF C_4_ PHOTOSYNTHESIS

C_4_ plants that carry C_4_ photosynthesis display higher CO_2_ assimilation rate then C_3_ plants under hot and semidry condition. Taniguchi et al. (https://doi.org/10.1002/tpg2.20095) analyzed the genus *Flaveria*, which is a model genus for studying C_4_ evolution, and showed that evolution of C_4_ photosynthesis progressed in a stepwise manner via an intermediate stage where cell‐specific expression of C_4_ cycle enzymes were gradually gained. They also published the whole‐genome sequences of C_3_, C_3_–C_4_ intermediate, C_4_–like, and C_4_ species of *Flaveria*, which is useful for future analysis to clarify the process of C_4_ evolution and also for engineering C_4_ photosynthesis in C_3_ plants.

## 14 TARGETING SORGHUM ANTHRACNOSE

The disease anthracnose caused by *Colletotrichum sublineola* constrains sorghum production on a global basis. The pathogen is extremely variable, meaning that there is a continual need to discover new sources of resistance to function against local isolates. Ahn et al. (https://doi.org/10.1002/tpg2.20097) tested 163 sorghum cultivars from Senegal for response against a mixture of Texas isolates and found that 49 were resistant. The study also identified eight single nucleotide differences across the genomes of these sorghum cultivars that can serve as prospective markers for alleles associated with resistance to anthracnose.

## 15 ANALYSES OF CANADIAN WHEAT MOLECULAR VARIATION

Canadian bread wheat cultivars generate close to 25 million tonnes of grain every year. Investigating allelic variation across this germplasm can explain why cultivars are genetically differentiated. Hargreaves et al. (https://doi.org/10.1002/tpg2.20099) report that allelic variation across Canadian wheat germplasm is in large part the result of breeding lines with shared attributes. Nonetheless, chromosomal sites have distinct patterns of variation. SNPs flanking major genes with large effects on traits are only sometimes associated with the traits. Alleles at homoeologous chromosomal regions have notably diverged between germplasm suggesting plant breeders selected for homoeologous loci. Finally, the germplasm has low allelic diversity across very long, recombination‐poor chromosomal regions, reducing its breeding potential.

## 16 SPINACH GENOME REVEALS EXTENSIVE REARRANGEMENTS AND RESISTANCE GENES

The limited contiguity of short‐read genome assemblies compromise annotation, and therefore genetic and genomic studies. Hulse‐Kemp et al. (https://doi.org/10.1002/tpg2.20101) report that a long‐read genome annotation identified and verified unreported resistance genes and transcription factors. It also revealed remnants of paleohexaploidy previously masked by extensive gene arrangements.

## 17 FIELD BASED HIGH‐THROUGHPUT PHENOTYPING DISCOVERS NOVEL PHENOMIC VARIATION AND UNDERLYING LOCI

Field based high‐throughput phenotyping using unoccupied aerial systems (UAS, also known as drones) is a newer approach to research and can complement genomic technology. Adak et al. (https://doi.org/10.1002/tpg2.20102) report that using temporal plant height data in maize (*Zea mays* L.) derived from drone images over growth reveal undiscovered phenotypic variation, especially in early growth stages, that has been overlooked via traditional phenotyping approaches. Plant height data belonging to different time points were found to have varying correlations with grain yield, likely due to vigor overcoming environmental stress. When temporal plant height data was used in a genome‐wide association study of the genomes to fields project (G2F), the majority of loci discovered were unique to specific time points, while a minority of loci were discovered in multiple time points. Genomic prediction results also showed that polymorphic markers throughout the maize genome had different effect sizes for plant height belonging to different time points. These results demonstrate temporal phenotype data of complex traits (e.g., plant height) should be considered as a phenomic source worth characterizing for use in maize breeding.

## 18 MICRORNAS FUNCTIONS IN SOYBEAN NODULES

Soybean is able to fix atmospheric nitrogen through the symbiotic relationship with nitrogen‐fixing rhizobia. The symbiotic nitrogen fixation occurs in a root organ called nodules where the host accommodates the bacteria. The soybean host supplies the bacteria with sugar and nutrients while the bacteria return  fixed nitrogen to the host. Fan et al. (https://doi.org/10.1002/tpg2.20103) report the differentially expressed microRNAs (miRNAs) in soybean roots and mature nodules. It was found that a portion of these miRNAs are involved in the acquisition of mineral nutrients that are related to the functions of symbiotic nitrogen fixation. Among these, miR399b, one highly induced miRNA in mature nodules, can enhance phosphate status of the soybean plant and consequentially enhances nitrogen fixation and overall plant growth.

## 19 RESISTANCE TO STEM RUST IN DURUM WHEAT

Many of the qualitative stem rust resistance genes in wheat became ineffective to specific races due to constantly emerging new virulent races. Moreover, the narrow genetic bases of stem rust resistance in durum wheat in many regions of the world encourages the use of a single resistance gene over a wide area that leads to a risk of complete crop loss due to potential epidemics. Megerssa et al. (https://doi.org/10.1002/tpg2.20105) identified durum wheat lines resistant against multiple single races at the seedling stage and in the field. Markers linked to the regions of several known stem rust resistance genes and novel regions that were consistently significant across multiple single races were discovered and they can be utilized in marker‐assisted selection and resistance gene pyramiding. The reliable markers can also be used to design high throughput markers which can be used for identifying other sources of resistance to stem rust, increasing the efficiency of future resistance breeding in durum wheat.

## 20 POSITIONAL CLONING IS OVERPOWERED BY STRUCTURAL VARIATION

Genomic structural variations are common among polyploids, but SNP‐based approaches to characterize genetic diversity are frequently insensitive to these variants. Taagen et al. (https://doi.org/10.1002/tpg2.20106) characterized a large deletion on wheat chromosome arm 5AS that had previously been misidentified as quantitative trait loci (QTL) harboring a causal gene for increased grain weight. Leveraging a fine‐mapping population, genomic data, phenotypic associations, early grain development expression profiles, and predicted gene function, the authors determined the QTL was a result of strong linkage disequilibrium with chromosome arm 5AS presence or absence. This study highlights that chromosome structural variation linkages can overpower the considerable resources required for positional cloning and discusses a more robust approach to gene fine‐mapping. The authors also present nine candidate genes on chromosome arm 5AS that may impact yield components, laying the foundation for identifying hidden variation of homoeolog dosage‐dependent and functionally redundant genes on the group five chromosome short arms. All analysis conducted is reproducible and publicly available as a learning resource at: https://github.com/etaagen/Taagen_2021_TPG.

## 21 HIGH‐RESOLUTION MARKERS DISSECT CAMELINA TRAITS

Genetic improvements are needed to advance *Camelina sativa* as an oilseed crop for sustainable production. Li et al. (https://doi.org/10.1002/tpg2.20110) found vast phenotypic variation in field trials in a worldwide collection of camelina accessions. They conducted whole‐genome resequencing to obtain high‐resolution, single nucleotide polymorphism markers for analyzing genomic diversity. Furthermore, by genome‐wide association studies of natural variation and linkage mapping in a recombinant inbred population, they identified quantitative trait loci and candidate genes that are associated with several important seed and agronomic traits. These genetic and genomic resources should assist in developing advanced camelina varieties by modern plant breeding and biotechnology.

